# Expressions of Type I and III Interferons, Endogenous Retroviruses, TRIM28, and SETDB1 in Children with Respiratory Syncytial Virus Bronchiolitis

**DOI:** 10.3390/cimb45020079

**Published:** 2023-02-02

**Authors:** Pier-Angelo Tovo, Silvia Garazzino, Francesco Savino, Valentina Daprà, Giulia Pruccoli, Maddalena Dini, Giacomo Filisetti, Elisa Funiciello, Ilaria Galliano, Massimiliano Bergallo

**Affiliations:** 1 Department of Pediatric Sciences and Public Health, University of Turin, Piazza Polonia 94, 10126 Turin, Italy; 2 Infectious Diseases Unit, Department of Pediatrics, Regina Margherita Children’s Hospital, Piazza Polonia 94, 10126 Turin, Italy; 3 Early Infancy Special Care Unit, Department of Pediatric Care, Regina Margherita Children’s Hospital, Piazza Polonia 94, 10126 Turin, Italy; 4 Pediatric Laboratory, Department of Pediatric Sciences and Public Health, University of Turin, Piazza Polonia 94, 10126 Turin, Italy

**Keywords:** respiratory syncytial virus, bronchiolitis, interferon, endogenous retroviruses, TRIM28, SETDB1

## Abstract

Interferons (IFNs) and IFN-stimulated genes (ISGs) play essential roles for the control of viral infections. Their expression in infants with respiratory syncytial virus (RSV) bronchiolitis is poorly defined. Human endogenous retroviruses (HERVs) represent 8% of our genome and modulate inflammatory and immune reactions. TRIM28 and SETDB1 participate in the epigenetic regulation of genes involved in the immune response, including IFNs and HERVs. No study has explored the expression of HERVs, TRIM28, and SETDB1 during RSV bronchiolitis. We assessed, through a PCR real-time Taqman amplification assay, the transcription levels of six IFN-I ISGs, four IFNλs, the pol genes of HERV-H, -K, and -W families, the env genes of Syncytin (SYN)1 and SYN2, and of TRIM28/SETDB1 in whole blood from 37 children hospitalized for severe RSV bronchiolitis and in healthy children (HC). The expression of most IFN-I ISGs was significantly higher in RSV+ patients than in age-matched HC, but it was inhibited by steroid therapy. The mRNA concentrations of IFN-λs were comparable between patients and age-matched HC. This lack of RSV-driven IFN-III activation may result in the defective protection of the airway mucosal surface leading to severe bronchiolitis. The expression of IFN-III showed a positive correlation with age in HC, that could account for the high susceptibility of young children to viral respiratory tract infections. The transcription levels of every HERV gene were significantly lower in RSV+ patients than in HC, while the expressions of TRIM28/SETDB1 were overlapping. Given the negative impact of HERVs and the positive effects of TRIM28/SETDB1 on innate and adaptive immune responses, the downregulation of the former and the normal expression of the latter may contribute to preserving immune functions against infection.

## 1. Introduction

Respiratory syncytial virus (RSV) is one of the most frequent causes of infection during the first 2 years of life, with approximately two thirds of children infected by the end of their first year [1]. Infants and young children may develop acute bronchiolitis upon RSV infection. Most patients do not require hospitalization; however, about 3% are hospitalized because of severe clinical pictures, sometimes with admission to a pediatric intensive care unit (PICU) and requiring the support of invasive mechanical ventilation [1,2]. Prematurity, younger age, and co-morbidity were identified as risk factors for increased morbidity and mortality [3,4]. However, nearly half of the children admitted to the PICU were previously healthy [2]. Severe RSV bronchiolitis may predispose children to childhood asthma [5,6]. With increasing age, RSV episodes decrease in severity and in adult severe manifestations occur only in individuals with previous pulmonary problems and in the elderly [7]. Most hypotheses about the particular susceptibility of infants to bronchiolitis are based on structural elements, such as small airway size, different innervation patterns, and lack of interalveolar pores and channels; however, biological factors also appear to be involved, such as an altered inflammatory response with increased mucus production, massive neutrophil accumulation, and prevalent Th2 responses in a genetically susceptible host [8].

Viral recognition elicits the production of interferons (IFNs), which in turn induce the transcription of IFN-stimulated genes (ISGs). These exert potent antiviral activities and modulate a large array of innate and adaptive immune responses. Three types of IFNs have been identified. Type I IFNs encompass several subtypes [9], which through their binding to a ubiquitous receptor (IFNAR) are able to induce a rapid transcription of thousands of genes [9,10]. The direct detection of type I interferons in biologic samples has proven to be challenging. Thus, expressions of ISGs are commonly used to calculate type I IFN signature scores [11,12]. In contrast to the type II IFN, which is mostly a lately produced T-cell cytokine, the type III interferon (IFN-III) is also involved in the early phases of the antiviral response. It encompasses four members, also referred to as lambdas (IFNλs). They bind to a unique receptor expressed by epithelial cells and a subset of immune cells [13]. IFNλs represent the front-line antiviral defense of the respiratory mucosa [14,15]; they have less inflammatory activity than type I IFNs and fine-tune their antiviral effects [16]. Given the potent IFN-driven antiviral functions, many viruses have developed mechanisms to escape their inhibitory effects [16,17,18], including RSV [19,20].

Human endogenous retroviruses (HERVs) constitute about 8% of the human genome. They originate from ancestral infections of somatic cells with subsequent stable integration into the DNA of germinal cells [21]. Following deletions, multiplications, and mutations, they cannot produce infectious virions. Some viral sequences are, however, transcribed, and a few encode proteins, such as Syncytin-1 (SYN1) [22] and Syncytin-2 (SYN2) [23], that are involved in crucial physiologic functions, such as placental syncytiotrophoblast formation and materno-fetal immune tolerance [24]. HERVs share the typical structure of retroviruses, with three major genes: group-associated antigens (gag), polymerase (pol), and envelope (env), flanked between two regulatory long terminal repeats (LTRs). HERVs can regulate the transcription of adjacent cellular genes [25,26]; their RNAs through retro-transposition may generate novel insertions into DNA or be sensed as non-self via pattern recognition receptors (PRRs) can elicit inflammatory and immune responses [26,27,28,29]. Some retroviral proteins can trigger autoimmunity [30,31], while others, such as syncytins, have intrinsic immunomodulatory properties [32,33,34,35]. Aberrant HERV expressions are associated with immune mediated and inflammatory diseases, supporting their pathogenetic role in these pathologies [25,30,31,36,37]. Exogenous viral infections can induce abnormal HERV transcription. These include human herpes simplex virus 1 [38,39] and 6 [39,40], varicella-zoster virus [40], Epstein–Barr virus [41,42], human cytomegalovirus [43], HIV [44,45], hepatitis B [46] and C [47] viruses, influenza virus [48], and SARS-CoV-2 [12,49,50,51]. Furthermore, IFNs and inflammatory cytokines lead to the independent and synergistic activation of retroviral sequences [52]. The impact of virus-induced aberrant HERV expressions on the antiviral immune response remains poorly understood. Protective effects have been reported [50,53], but growing evidence suggests a negative action on viral control and disease progression [33,34,49,51,54,55,56].

HERV expression may be modulated by environmental factors through epigenetic mechanisms, such as DNA methylation and histone modifications. Krüppel-associated box domain zinc finger proteins (KRAB-ZFPs) represent the largest family of transcription regulators in our genome. The tripartite motif-containing 28 (TRIM28), also called KAP1 or TIF1-β, is a nuclear corepressor of KRAB-ZFPs [57]. SET domain bifurcated histone lysine methyltransferase 1 (SETDB1), also called ESET, is a histone H3K9 methyltransferase that participates with TRIM28 and KRAB-ZFPs to form heterochromatin [58]. TRIM28 and SETDB1 were uncovered mainly through investigations on host factors regulating the transcription of endogenous retroviruses [59,60]. Recent studies have, however, drawn attention to the crucial roles of TRIM28 and SETDB1 in the epigenetic control of the immune response [60,61,62,63], including the antiviral response and IFN production [64,65,66].

Despite these premises, the importance of IFN signature scores in infants hospitalized with severe RSV bronchiolitis remains elusive. Furthermore, in light of the potential cross-talks between RSV infection, HERVs, and TRIM28/SETDB1, no data on their expressions in infected patients are available. To obtain targeted information on the relevance of these variables during severe RSV bronchiolitis, we compared the transcriptional levels of six IFN-I ISGs and of type III IFNs, of HERV sequences, in particular of env genes of SYN1 and SYN2, and of pol genes of HERV-H, -K, and -W (the three retroviral families most widely studied), as well as of TRIM28 and SETDB1 in children hospitalized for acute RSV bronchiolitis versus healthy children (HC).

## 2. Materials and Methods

### 2.1. Study Populations

Two groups of children were enrolled in the study. Group A included children who were admitted at the Regina Margherita Children’s Hospital, Turin, Italy, with acute bronchiolitis due to RSV infection. Group B included HC tested at the same hospital for various reasons, such as planned surgical interventions and the exclusion of genetic, metabolic, endocrinological, or nephrological diseases, whose laboratory examinations, including white blood cell count and inflammatory markers, were all within normal ranges for age and whose results were all within normal limits. Subjects with any confirmed or suspected diseases associated with abnormal IFN production and/or HERV expression were excluded from the study. Exclusion criteria included children with confirmed or suspected immune defects (e.g., primary immunodeficiencies, born to HIV+ mothers), cancers, viral coinfections, autoimmune disorders [31], food allergies [37], prematurity [67], or autism spectrum disorder [68,69].

### 2.2. Total RNA Extraction

Total RNA was extracted from whole blood using the automated extractor Maxwell following the RNA Blood Kit protocol without modification (Promega, Madison, WI). This kit provides treatment with DNase during the RNA extraction process. To further exclude any contamination of genomic DNA, RNA extracts were directly amplified without reverse transcription to validate the RNA extraction protocol. RNA concentration and purity were assessed via traditional UV spectroscopy with absorbance at 260 and 280 nm. The nucleic acid concentration was calculated using the Beer–Lambert law, which predicts a linear change in absorbance with concentration. The RNA concentration range was within manufacturer specifications for the NanoDrop (Thermo Fisher Scientific, Waltham, MA USA). UV absorbance measurements were acquired using 1 µL of RNA sample in an ND-1000 spectrophotometer under the RNA-40 settings at room temperature (RT). Using this equation, an A260 reading of 1.0 is equivalent to ~40 µg/mL single-stranded RNA. The A260/A280 ratio was used to define RNA purity. An A260/A280 ratio of 1.8/2.1 is indicative of highly purified RNA. The RNAs were stored at −80° until use.

### 2.3. Reverse Transcription

Four hundred nanograms of total RNA was reverse-transcribed with 4 μL of buffer 5X, 4.8 μL of MgCl2 25 mM, 2 μL ImpromII (Promega), 1 μL of RNase inhibitor 20 U/L, 0.4 μL random hexamers 250 μM (Promega), 2 μL mix dNTPs 100 mM (Promega), and dd-water in a final volume of 20 μL. The reaction mix was carried out in a GeneAmp PCR system 9700 Thermal Cycle (Applied Biosystems, Foster City, CA, USA) under the following conditions: 5 min at 25 °C, 60 min at 42 °C, and 15 min at 70 °C for the inactivation of the enzyme. The cDNAs were stored at −20 °C until use.

### 2.4. Transcription Levels of IFNs, ISGs, TRIM28, SETDB1, Pol Genes of HERV-H, -K, and -W, and Env Genes of SYN1 and SYN2 via Real-Time PCR Assays

GAPDH was chosen as the reference gene in all determinations, being one of the most stable among the reference genes and already used in our previous studies [12,36,37,47,69].

Relative expression of transcription levels of IFN-I ISGs (IFI27, IFI44L, ISG15, IFIT1, RSAD2, and SIGLEC) [11], IFN-III (IFN-λ1, IFN-λ2, IFN-λ3, and IFN-λ4) [13], TRIM28, and SETDB1, as well as of HERV-H-pol, HERV-K-pol, HERV-W-pol, SYN1-env, and SYN2-env was achieved as previously described in detail [12,54], using the primers and probes reported in Table 1. Briefly, 40 ng of cDNA was amplified in a 20 μL total volume reaction, containing 2.5 U goTaQ MaterMix (Promega), 1.25 mmol/L MgCl2, 500 nmol of specific primers, and 200 nmol of specific probes.

All of the amplifications were run in a 96-well plate at 95 °C for 10 min, followed by 45 cycles at 95 °C for 15 s and at 60 °C for 1 min. Each sample was run in triplicate. Relative quantification of target gene transcripts was performed according to the 2^-ΔΔCt^ method [70]. Briefly, after normalization of the PCR result of each target gene with the housekeeping gene, the method included additional calibration using the expression of the target gene evaluated in a pool of healthy controls. The results, expressed in arbitrary units (called relative expression, RE), show the variations in target gene transcripts relative to the standard set of controls. Since we measured Ct for every target in all samples, we argue that our methods were suitable for HERVs, IFN signatures, TRIM28 and SETDB1 detection, and quantifications.

All analyses were performed in a laboratory of biosafety level 2 (BSL-2), according to the NIH [71] and WHO [72] guidelines.

### 2.5. Statistical Analysis

A one-way ANOVA test was used to compare the transcriptional levels of IFN-I ISGs, IFN-III, TRIM28, SETDB1, HERV-H-pol, HERV-K-pol, HERV-W-pol, SYN1-env, and SYN2-env, between Group A1 and Group A2 patients and HC. The Mann–Whitney test was used to compare the transcriptional levels of each IFN-I ISG, IFN-III, TRIM28, SETDB1, HERV-H-pol, HERV-K-pol, HERV-W-pol, SYN1-env, and SYN2-env between each group of children with each other. The Spearman correlation test was used to evaluate the correlations between age and mRNA concentrations of each target gene. Statistical analyses were conducted using the Prism software (GraphPad Software, La Jolla, CA, USA). In all analyses, *p* < 0.05 was taken to be statistically significant.

## 3. Results

### 3.1. Study Populations

Group A included 37 children hospitalized for acute RSV bronchiolitis. Their demographics and clinical features are reported in Table 2. Three RSV+ infants with Influenza A coinfection and one with pre-term delivery were excluded from the study.

Ten patients had comorbidities: two had asthma, one had metabolic disorders, two had congenital malformations, two had bacterial coinfections, one had neuromuscular disease, one was late pre-term, and one had neuropsychomotor disorder. Three patients had complications: one had bacterial pneumonia, one had cutaneous vasculitis, and one had post-infective anemia. Supplemental oxygen was administered to 31 patients. One patient had been admitted to the ICU. High markers of systemic inflammation, such as C-reactive protein (CRP), erythrocyte sedimentation rate (ESR), and procalcitonin (PCT) levels, were found in 10 (27%) patients.

Patients were subdivided into two subgroups: Group A1 was on steroid treatment, Group A2 was without such therapy (Table 3). No specific criteria were used to select children who underwent steroid treatment; this was the subjective choice of individual pediatricians.

At follow-up, all patients recovered from the acute illness.

As detailed in Table 3, control children encompassed four subgroups of subjects (Group B1, B2, B3, and B4), based on the tests performed.

### 3.2. Influence of Age on IFN Signature Scores and on Expression Levels of HERVs, TRIM28, and SETDB1

**Figure 1:**
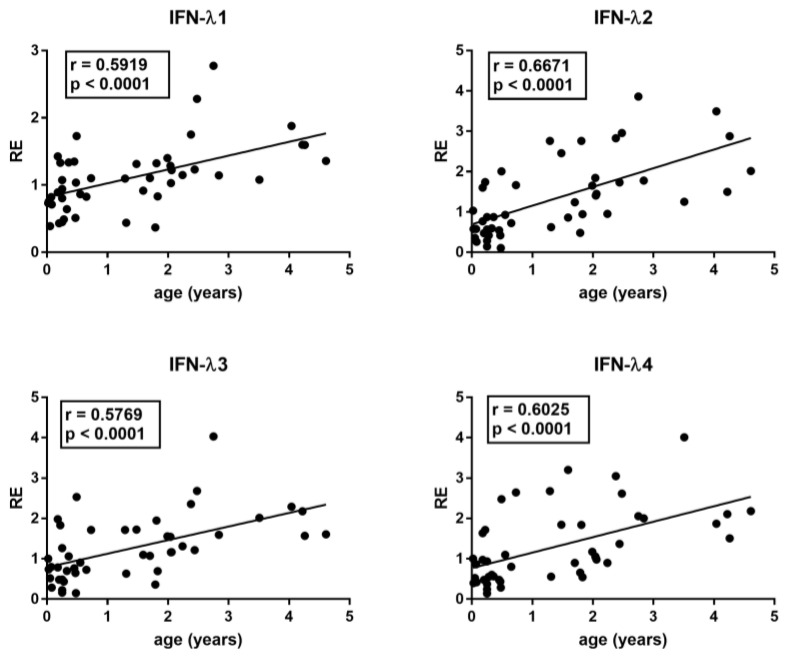
Correlations between age and transcription levels of IFNλ1, IFNλ2, IFNλ3, and IFN-λ4 in whole blood from 46 healthy children of 0–5 years of age. RE: relative expression. Circles show the mean of three individual measurements. Line: linear regression line. Statistical analysis: Spearman correlation test.

The median age differed between RSV patients and control groups. In particular, Group A children were significantly younger than Group B1, B2, B3, and B4 children (*p* < 0.0001). However, with the exception of IFN-III, there was no significant correlation between age and the transcriptional levels of each target gene in the control groups, even if there was a trend of negative correlation for TRIM28 and SETDB1. In contrast, there was a significantly positive correlation between the age and the mRNA levels of IFN-λ1, IFN-λ2, IFN-λ3, and IFN-λ4 in HC (Figure 1).

### 3.3. Type I IFN Signature

**Figure 2:**
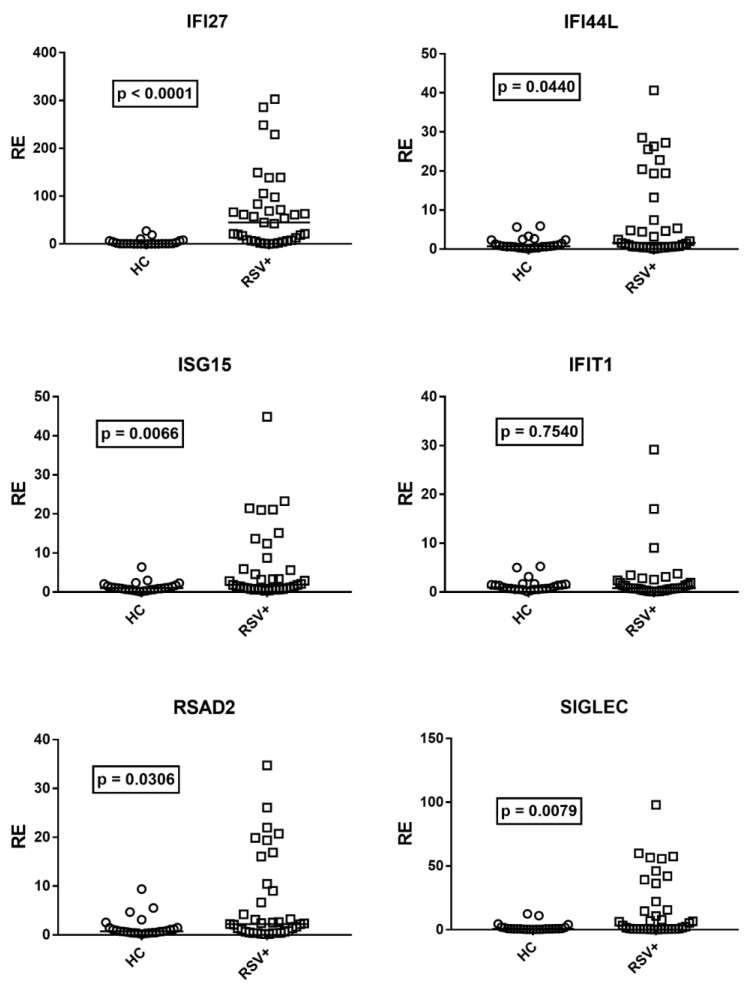
Expression of type I interferon stimulated genes (ISGs) in whole blood from 37 children with acute RSV bronchiolitis (RSV+) and 25 age-matched healthy children (HC). RE: relative expression. Circles and squares show the mean of three individual measurements; horizontal lines show the median values. Median values and interquartile range 25–75% of ISGs: IFI27: HC 0.55, 0.31–4.60; RSV+ 44.98, 7.90–83; IFI44L: HC 0.72, 0.54–2.36; RSV+ 1.59, 057–13.23; ISG15: HC: 1.02, 0.62–1.50; RSV+ 1.85, 0.78–5.89; IFIT1: HC 0.97, 0.59–1.49; RSV+ 0.79, 0.48–1.91; RSAD2: HC 0.76, 0.46–1.45; RSV+ 2.21, 0.66–9.02; SGLEC: HC 0.73, 0.48–1.63; RSV+ 3.43, 0.68–22.05. Statistical analysis: Mann–Whitney test was used to compare values of each group of children with each other.

Although there was no correlation between age and expressions of IFN-I ISGs in HC, for analogy with IFN-III, we compared the median transcription levels of IFN-I ISGs in children with RSV bronchiolitis with the subgroup of 25 age-matched HC (median age and 25–75% IQR: RSV+ patients: 0.2, 0.1–0.3; HC: 0.3, 0.2–0.6, *p* = 0.11). Figure 2 shows that the mRNA levels of IFN-I ISGs were significantly higher in RSV+ patients than in HC for all ISGs, with the exception of IFIT1.

No significant differences between males and females were found in the transcription levels of any ISG, both in HC and in RSV+ patients (Supplementary Figures S1 and S2).

**Figure 3:**
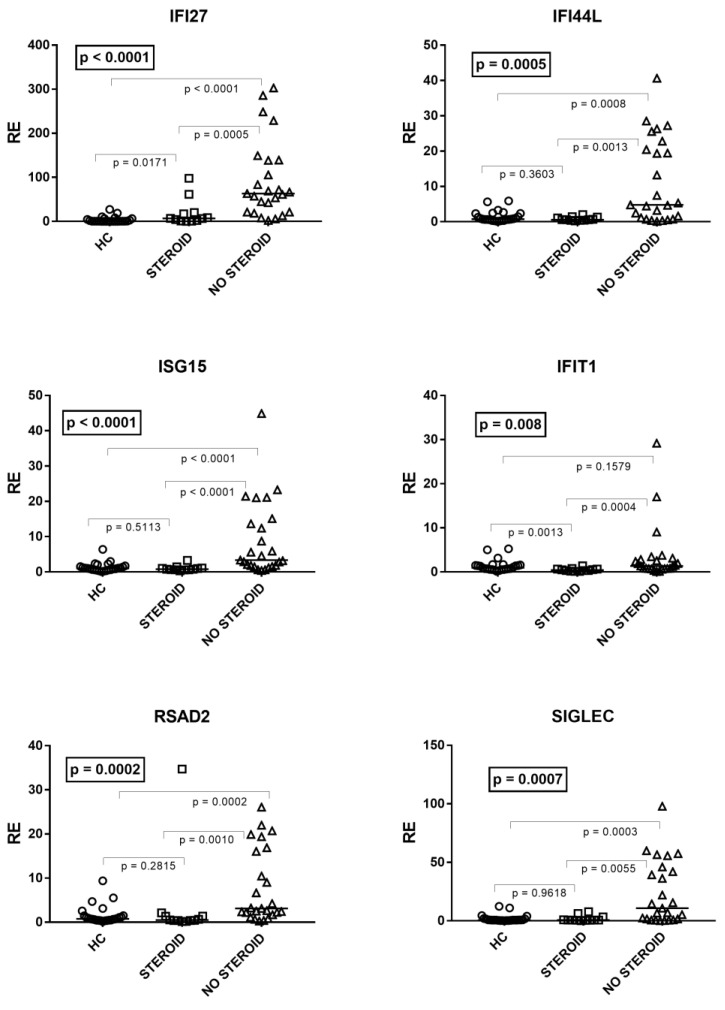
Expression of type I interferon stimulated genes (ISGs) in whole blood from 25 age-matched healthy children (HC), 12 children with acute RSV bronchiolitis on steroid treatment (Group A1), and 25 untreated patients (Group A2). RE: relative expression. Horizontal lines show the median values; squares, triangles, and circles show the mean of three individual measurements. Median values and interquartile range 25%–75% of six ISGs (values of HC are reported above): IFI27: Group A1 (on steroids) 6.74, 1.90–17.78; Group A2 (untreated) 63.32, 21.13–138.40; IFI44L: Group A1 0.58, 0.43–1.12; Group A2 4.78, 1.12–20.44; ISG15: Group A1 0.77, 0.59–1.05; Group A2 3.35, 1.81–13.66; IFIT1: Group A1 0.41, 0.28–0.66; Group A2 1.36, 0.75–2.82; RSAD2: Group A1 0.47, 0.39-1.36; Group A2 3.11, 2.19–16.04; SIGLEC: Group A1 0.73, 0.59–1.58; Group A2 10.76, 1.67–42.09. Statistical analysis: The transcription levels of each target between the three groups of children were compared using the one-way ANOVA test. The transcription levels of each target between each group of children with each other were compared using the Mann–Whitney test.

As illustrated in Figure 3, by comparing patients on steroid treatment (Group A1), untreated patients (Group A2), and HC, one-way ANOVA analysis showed significant differences in the mRNA levels of every IFN-I ISG between the three groups of children. In particular, the median transcriptional levels of IFN-I ISGs were significantly higher in untreated patients vs. HC for all genes except IFIT1. The levels of transcripts were significantly lower in treated patients vs. untreated patients for every ISG, whereas in the former vs. HC they remained significantly higher for IFI27, comparable to IFI44L, ISG15, RSAD2, and SIGLE, and reduced for IFIT1 (Figure 3).

### 3.4. Type III IFNs

**Figure 4:**
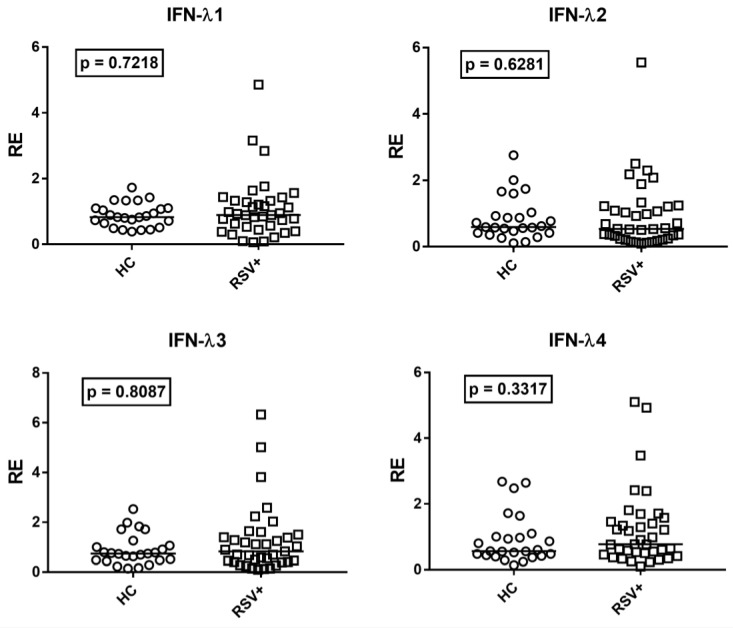
Expression of type III interferons in whole blood from 37 children with acute RSV bronchiolitis (RSV+) and 25 age-matched healthy children (HC). RE: relative expression. Circles and squares show the mean of three individual measurements; horizontal lines show the median values. Median values and interquartile range 25–75% of IFN-IIIs: IFN-λ1: HC 0.83, 0.64–1.10; RSV+ 0.89, 0.53–1.32; IFN-λ2: HC 0.59, 0.42–0.93; RSV+ 0.54, 0.25–1.21; IFNλ3: HC 0.74, 0.48–1.06; RSV+ 0.83, 0.41–1.39; IFNλ4: HC 0.56, 0.44–1.00; RSV+ 0.77, 0.50–1.46. Statistical analysis: Mann–Whitney test was used to compare values of each group of children with each other.

Given the positive correlations between age and IFN-III levels, the transcriptional levels of every IFN-λ in RSV-infected children were compared to the subgroup of the 25 age-matched HC. The transcription levels of all IFN-λs were similar in RSV-infected patients and age-matched HC (Figure 4).

By comparing Group A1 patients (on steroid therapy) vs. Group A2 patients (untreated), no significant difference emerged in the IFN-III transcript levels. Median values and interquartile range 25%-75% of IFN-IIIs: IFN-λ1: Group A1 0.81, 0.54–1.02; Group A2 0.93, 0.53-1.44; (*p* = 0.2149); IFN-λ2: Group A1 0.54, 0.23–0.77; Group A2 0.54, 0.34–1.22; (*p* = 0.4909); IFN-λ3: Group A1: 0.77, IQR 0.45–1.20; Group A2 0.90, 0.41–1.61; (*p* = 0.6426); IFN-λ4: Group A1 0.83, 0.44–1.27; Group A2 0.77, 0.55–1.46 (*p* = 0.5322).

No significant differences between genders were observed for IFN-III both in HC and RSV+ patients (Supplementary Figures S3 and S4).

### 3.5. Expressions of HERV-H-Pol, HERV-K-Pol, HERV-W-Pol, SYN1-Env, and SYN2-Env

**Figure 5:**
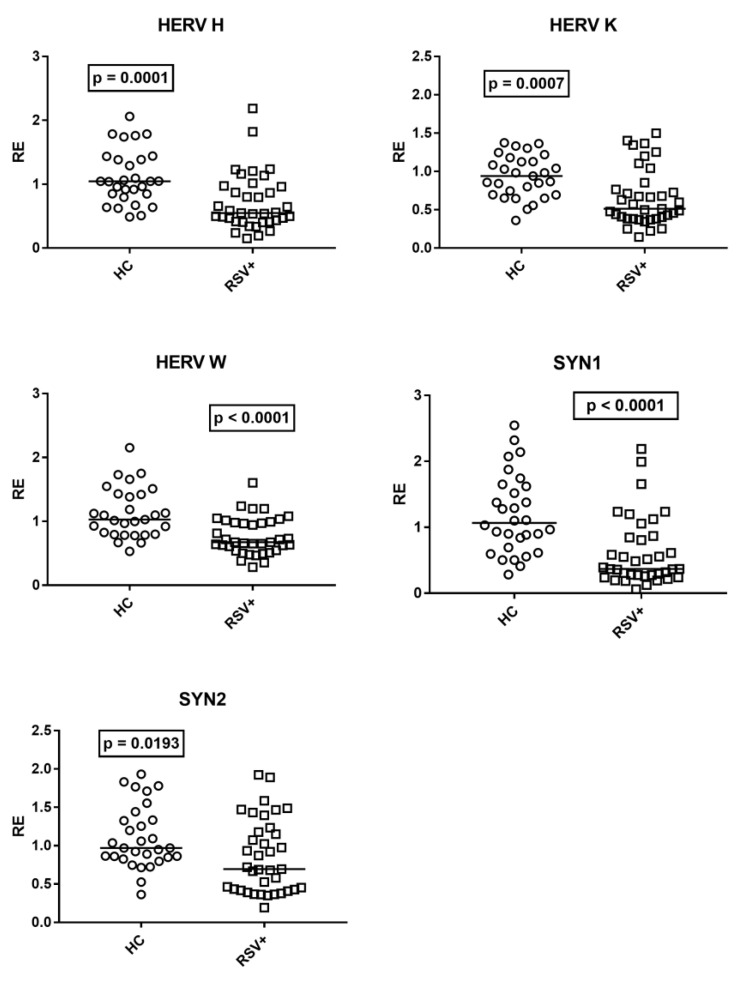
Transcription levels of pol genes of HERV-H, HERV-K, and HERV-W, and of env genes of syncytin (SYN)1 and syncytin (SYN)2 in whole blood from 37 children with acute RSV bronchiolitis (RSV+), from 29 healthy children (HC) for HERV-pols, and 30 HC for SYN1-env and SYN2-env. RE: relative expression. Circles and squares show the median of three individual measurements; horizontal lines show the median values. Median values and interquartile range 25–75%, HERV-H-pol: HC 1.05, 0.85–1.39; RSV+ 0.55, 0.42–0.96; HERV-K-pol: HC 0.94, 0.70–1.13; RSV+ 0.51, 0.39–0.77; HER-W-pol: HC 1.03, 0.80–1.42; RSV+ 0.67, 0.55–0.98; SYN 1-env: HC 1.07, 0.73–1.60; RSV+ 0.37, 0.28–0.85; SYN2-env: HC 0.97, 0.85–1.33; RSV+ 0.69, 0.43–1.18. Statistical analysis: Mann–Whitney test was used to compare values of each group of children with each other.

The medians of the transcription levels of HERV-H-pol, HERV-K-pol, and HERV-W-pol, as well as of SYN1-env and SYN2-env, were significantly lower in children with RSV bronchiolitis as compared to HC (Figure 5).

No significant differences between genders were observed in the transcription levels of HERVs both in HC and RSV+ patients (Supplementary Figures S5 and S6).

No difference was found between patients with (Group A1) and without (Group A2) steroid treatment. Median values and interquartile range 25–75%, HERV-H-pol: Group A1 0.54, 0.46–0.84; Group A2 0.56, 0.41–0.97; (*p* = 0.9618); HERV-K-pol: Group A1 0.59, 0.44–0.84; Group A2 0.50, 0.38–0.77; (*p* = 0.6892); HERV-W-pol: Group A1 0.70, 0.52–1.00; Group A2 0.66, 0.61–0.97; (*p* > 0.9999); syncytin 1-env: Group A1 0.55, 0.33–1.24; Group A2 0.34, 0.26–0.81; (*p* = 0.2273); syncytin 2-env: Group A1 0.81, 0.43–1.24; Group A2 0.68, 0.42–1.15; (*p* = 0.9109).

### 3.6. Expressions of TRIM28 and SETDB1

**Figure 6:**
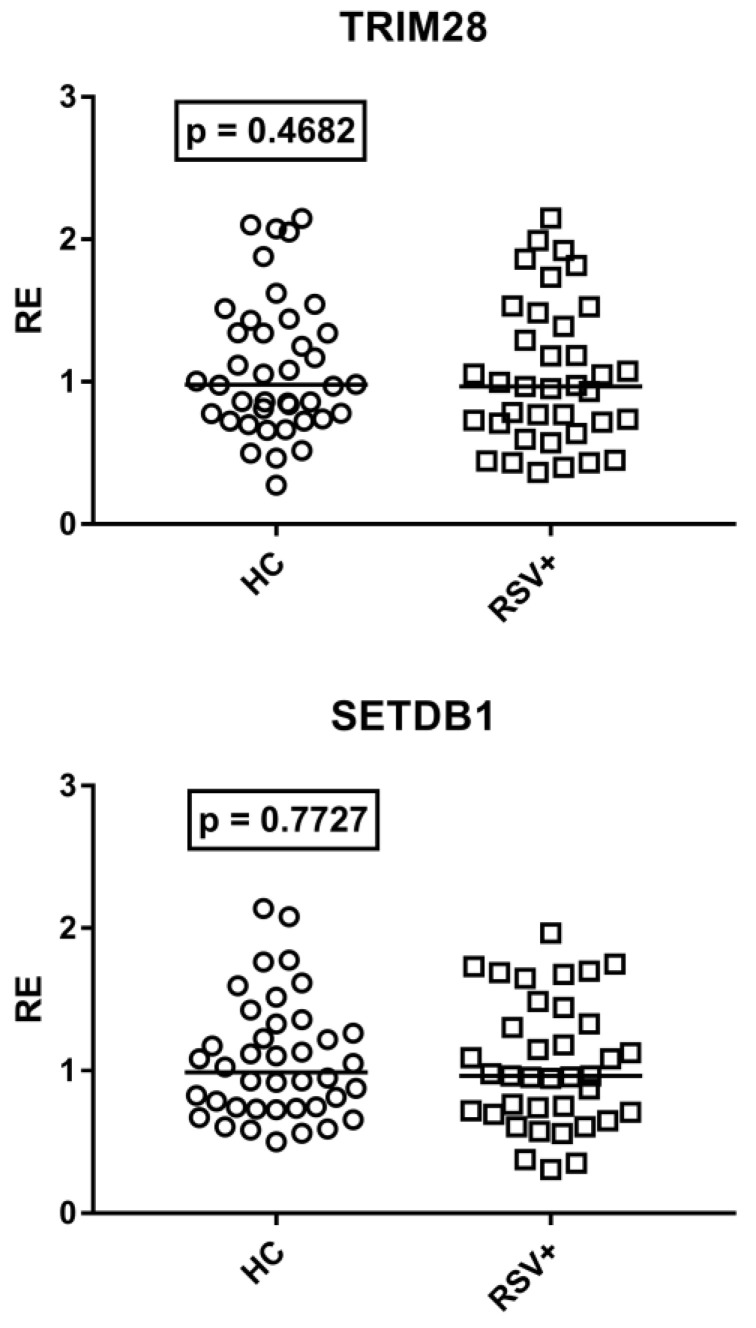
Expression of TRIM28 and SETDB1 in whole blood from 40 healthy children (HC) and 37 children with acute RSV bronchiolitis. RE: relative expression. Circles and squares show the median of three individual measurements; horizontal lines show the median values. Median values and interquartile range 25–75%: TRIM28: HC 0.98, 0.77–1.36; RSV+ 0.97, 0.71–1.39; SETDB1: HC 0.99, 0.74–1.28; RSV+ 0.96, 0.71–1.33. Statistical analysis: Mann–Whitney test was used to compare values of each group of children with each other.

As detailed in Figure 6, TRIM28 and SETDB1 expression levels were comparable between RSV-infected patients and HC.

No difference was found between patients with (Group A1) and without (Group A2) steroid treatment. Median values and interquartile range 25–75%: TRIM28: Group A1 0.87, 0.70–1.53; Group A2 0.97, 0.71–1.18; (*p* = 0.8604); SETDB1: Group A1 1.03, 0.75–1.50; Group A2 0.96, 0.72–1.30; (*p* = 0.5753).

TRIM28 and SETDB1 expressions did not show any significant difference between males and females in HC and RSV+ patients (Supplementary Figures S7 and S8).

## 4. Discussion

The key role of IFNs in the control of viral infections is widely recognized, although their response is qualitatively and quantitatively virus-specific, and their production may vary during the course and the severity of the disease. Type I and type III IFNs are thought to be the main players in controlling the early steps of viral spread. Type I IFN production was found to be impaired in RSV-infected human airway epithelial cells [73]. RSV-nonstructural protein (NS)1, -NS2, and envelope G glycoprotein inhibit IFN-I synthesis [74,75,76,77]. The suppressor of the cytokine signaling (SOCS) family, through a feedback loop, inhibits the IFN-I-dependent antiviral signaling pathway, and NS1 and NS2 proteins upregulate SOCS1 and SOCS3 [78]. All of these findings suggest that RSV relies upon IFN-I downregulation to evade the early antiviral response. Our results, however, highlight that IFN-I signature scores were significantly higher for most ISGs in children hospitalized for acute RSV bronchiolitis without steroid treatment, as compared to age-matched HC. These findings suggest that the RSV infection is actually able to trigger a significant type I IFN synthesis, although the magnitude of this response may be lower than in other acute viral infections. For instance, RSV elicited a type I IFN production in the nasal mucosa of infants, but at significantly lower amounts as compared to the robust influenza virus response [73,79]. The result may be limited to the protection of the spread of RSV to lower airways. The RSV-driven IFN-I production was reduced during the neonatal period in an animal model [80] and in newborns and young children [20,81]. However, we did not observe any significant influence of the age on the expression of IFN-I ISGs in infants and pre-school healthy children. Furthermore, we found a higher expression of IFN-I ISGs by comparing RSV+ patients with age-matched HC.

The pharmacological approach in children with bronchiolitis is still debated. Given their strong anti-inflammatory activity and the lack of side effects in the short term, the administration of steroids is not unusual. However, since there is no evidence that systemic or inhalation steroids provide a significant benefit for bronchiolitis [82,83], their administration is discouraged by several guidelines [84,85]. Nevertheless, these continue to be administered for the management of RSV bronchiolitis in pediatric wards [86,87]. In line with the steroid-induced suppression of IFNs [88,89], our data document that patients on steroid treatment had a significantly impaired transcription of IFN-I ISGs as compared with untreated patients. This provides a biological basis for the more prolonged viral shedding in patients treated with steroids [90], and further supports that these should not be used in RSV bronchiolitis treatment.

The type III IFN machinery seems especially implicated in the protection of mucosal surfaces from viral attacks [14,15]. A first important point emerging from our results is the positive correlation between age and the transcription levels of every IFN-λ in HC. Since IFN-III protects the respiratory and gastrointestinal tract epithelial cells from viral infections [91], the impaired expression of IFN-III in the early period of life may contribute, at least in part, to the typical, high susceptibility of infants and pre-school children to viral infections of airways and the gastrointestinal tract. The second point is the similar concentration of all IFN-λ mRNAs in children with severe RSV bronchiolitis and in age-matched HC. This suggests that the IFN-III pathway remained unstimulated in RSV-infected children with disease progression. TRIM28/SETDB1 and steroids exert relevant regulatory activities in the induction of IFNs, but their expressions remained comparable between RSV-infected patients and HC as well as between patients with and without steroid therapy. IFN-III participates in the first-line defense against RSV via the retinoic-acid-inducible gene-I-(RIG-I)-dependent pathway [92,93]. However, RSV-NS1, -NS2, G proteins, and RSV-driven high concentrations of SOCS-1 and SOCS3 are able to inhibit not only the expression of IFN-I, but also of IFN-III [77,78,94,95]. The suppression of IFN-III was also documented via the RSV-induced elevated levels of prostaglandin 2 [96], and the activation of the epidermal growth factor receptor [97] and of Rab5a [98]. Moreover, the type III IFN response was significantly lower in children with RSV bronchiolitis as compared to children with influenza infection [73]. Therefore, the impaired expression of IFN-III in the first period of life, along with the ability of RSV to downregulate its production, may account for the lack of enhanced transcription levels of IFN-III in our patients and justify the development of severe pulmonary complications. In this context, it must be underlined that blood samples were collected after a long time from symptom onset, when the role-effect of IFN-III could be exhausted. High concentrations of IFN-λ1 were actually found in nasal airway secretions at the onset of acute RSV respiratory illness [99]. Furthermore, we did not assess the RSV strain, which may impact the IFN-I and IFN-III expression profiles and the magnitude of the ISG response [100,101].

Present results highlight, for the first time, that in children hospitalized for acute RSV bronchiolitis, the transcriptional levels of pol genes of HERV-H, -K, and -W, as well as of env genes of SYN1 and SYN2 were significantly lower than in HC. The origin and the clinical significance of such reduced HERV transactivation remain to be elucidated. TRIM28 and SETDB1 are considered to be potent co-repressors of HERV transcription [57,58,59,60]. However, their mRNA levels were similar to those of HC, suggesting that the HERV downregulation was not sustained by the abnormal expression of TRIM28 or SETDB1. Steroids can modulate the activation of retroviral elements [102,103], but we did not find any differences in HERV transcripts between treated and untreated patients. Inflammatory cytokines can stimulate the activation of retroviral sequences [52]. Increased inflammatory markers or alterations in white blood cell count were, however, observed only in a minority of patients. Therefore, the RSV induces strong, local, but poor systemic inflammation in children with severe bronchiolitis. Recently, the nuclear accumulation of RSV NS1 and the matrix (M) protein has been linked to global changes in host gene transcription [104,105]. Whether this also induces similar alterations in retroviral sequences remains to be investigated.

HERVs are involved in the host defense against viral infections and may trigger immune-mediated damages [25,28]. For instance, a HERV-K envelope protein inhibits the release of cytokines [54] and counteracts tetherin-mediated antiviral activity [55]. Since HERV-K elements are highly polymorphic in the human population, interindividual variations among HERV-K-env genes may result in different immune responses against the same viral infection [55,106]. A HERV-W envelope protein exerts potent pathogenic action through CD14/TLR4 stimulation [27,30]. SYN1 antagonizes antiviral responses and increases virus-induced inflammation [32,35,56,107], while SYN2 vigorously suppresses the T cell functions [34]. In general, the lower expression of HERVs in our patients may thus mirror a positive effect for the host deriving from their downregulation. These low HERV transcription levels seem antithetical to their increased activation, usually elicited by exogenous viral infections. An enhanced HERV expression was noticed, however, in chronic infections, such as those due to herpesviruses [38,39,40,41,42,43], HIV [44,45], and hepatitis viruses [46,47], or in vitro due to influenza viruses [48]. Upon an acute infection, such as SARS-CoV-2 infection, children with mild/moderate symptoms showed enhanced HERV transcription, whereas those with severe clinical pictures and a long duration of disease had significant declines in their mRNA concentrations, even below the normal values [12]. The present patients were all hospitalized for severe bronchiolitis and blood samples were collected after a long time from the beginning of the infection. Taken together, these findings suggest that reduced HERV expressions might characterize a severe, prolonged course of acute viral infections. This could be due to an exhaustion of the virus-driven stimulatory mechanisms and/or the upregulation of specific inhibitory checkpoints. For instance, the sterile alpha motif and HD-domain-containing protein 1 (SAMHD1) are able to block inflammatory responses and the activation of retroviruses during viral infections [108]. Based on this, children with mild forms of RSV infection and/or tested in the first days of illness could exhibit normal or increased levels of HERV mRNAs, with low concentrations of transcripts being a feature of a progressive acute disease.

The final biologic effects of HERVs on cell homeostasis are expected to be mediated by proteins. We assessed their transcriptional profiles, not their protein coding capacities. This is a limitation of our study, which, however, does not undermine the potential impact of HERVs on the evolution of RSV infection. As non-coding regulatory elements, HERVs can act as promoters and enhancers of cellular genes [25,26]. Their RNAs may be reintegrated everywhere into the DNA or recognized as non-self by viral RNA receptors triggering innate and adaptive immune responses [26,27,28,29].

RSV infection may induce epigenetic changes with possible clinical consequences [109,110]. Many members of the large family of TRIM proteins contribute to blocking viral replication [111] and growing data shed light on the pivotal roles of TRIM28/SETDB1 in epigenetic control of the immune response, including antiviral effects and T cell differentiation and functions [60,61,62,63,64,65,66]. The normal mRNA concentrations of TRIM28/SETDB1 that emerged in our patients, however, suggest that they are not key players in RSV-driven epigenetic variations. On the other hand, the activation of TRIM28/SETDB1 is a highly dynamic process that is removed rapidly by specific deconjugating proteases [112,113]. The prolonged time interval between symptom onset and blood collection could thus have influenced their transcription levels.

## 5. Conclusions

Our study shows the enhanced expression of many IFN-I ISGs in children hospitalized for severe RSV bronchiolitis without steroid treatment. The significant suppression of ISGs following the administration of steroids further points out that these should not be used in RSV bronchiolitis. The reduced expression of IFN-λs in the first period of life in HC and the lack of their activation in patients with disease progression may mirror an inadequate IFN-III protection of the airway mucosa surface that exposes RSV-infected infants to a high risk of pulmonary involvement. Type III IFN is of therapeutic interest due to its low proinflammatory profile. Our data support its potential use in RSV-infected at-risk infants to prevent the development of respiratory complications. The positive correlation between age and the expression of IFN-III may account for the high frequency of viral infections of the respiratory and gastrointestinal tracts in infants and pre-school children. The significantly impaired HERV transcription in children with RSV bronchiolitis was an unexpected feature. Its underlying biochemical mechanisms and clinical consequences remain to be elucidated. It does not seem imputable to inhibitory effects of corticosteroids or to alterations in TRIM28/SETDB1 repressors. The potential negative impact of HERV activation on the immune system is increasingly recognized. Its downregulation in children with severe RSV bronchiolitis may be a biomarker of disease progression, representing an attempt to limit its negative effects on the host defensive mechanisms. In contrast, given the essential role of TRIM28/SETDB1 in innate and adaptive immune responses, its preserved expression may contribute to maintaining effective immune protection against infection.

## Figures and Tables

**Table 1:** Primers and probes used to assess the transcription levels of type I interferon stimulated genes, type III interferon genes, pol genes of HERV-K, -W, and –H, env genes of syncytin 1 and syncytin 2, TRIM28, and SETDB1.

Name	Primer/Probe	Sequence
Type I interferon stimulated genes		
IFI27	Forward	TGTCATTGCGAGGTTCTACTAGCT
	Reverse	CCCCTGGCATGGTTCTCTT
	Probe	6FAM-CCTGCCCCTCGCCCTGCA-TAMRA
IFI44L	Forward	GTGACTGGCCAAGCCGTAGT
	Reverse	CACACAACATAAATGGCAGAGATTT
	Probe	6FAM-TCTGATATCACCAGCATAACCGAGCGG-TAMRA
IFIT1	Forward	TGGCTGACTTCACCTAGCTCACT
	Reverse	CATGGACTGGCCAGAACCA
	Probe	6FAM-CGTAGCGCCACAGCCAGACTCCC-TAMRA
ISG15	Forward	TGGCGGGCAACGAATT
	Reverse	GGGTGATCTGCGCCTTCA
	Probe	6FAM-CCTGAGCAGCTCCATGTCGGTGTC-TAMRA
RSAD2	Forward	GAGGGCCAGATGAGACCAAA
	Reverse	GTGAAGTGATAGTTGACGCTGGTT
	Probe	6FAM-AGGACCCTCCTCTGCCCACCACC-TAMRA
SIGLEC1	Forward	AGGGAGACTGGGAAATGTAGTTTTTA
	Reverse	ATTCCCAACAATGTCAAAAGTCTCA
	Probe	6FAM-AGTCCAGAGGACATTTGGAATTGGAC-TAMRA
Type III interferon		
IFN-λ1	Forward	GAGGCATCTGTCACCTTC
	Reverse	GGTTGACGTTCTCAGACA
	Probe	6FAM-ACCTCTTCCGCCTCCTCACG-BHQ1
IFN-λ2	Forward	GCCACATAGCCCAGTTCAAG
	Reverse	TCCTTCAGCAGAAGCGACTC
	Probe	6FAM-CTGTCTCCACAGGAGCTGCAGGCC-BHQ1
IFN-λ3	Forward	TCACCTTCAACCTCTTCC
	Reverse	GAAGGGTCAGACACACAG
	Probe	6FAM-TGGCAACACAATTCAGGTCTCG-BHQ1
IFN-λ4	Forward	CCTTCTACAGGGAAGAGAC
	Reverse	CTGGTAACCACACAAGGA
	Probe	6FAM-CAGTTCTCCAGGAAGCCACGATA-BHQ1
HERV-K *pol*	Forward	CCACTGTAGAGCCTCCTAAACCC-
	Reverse	TTGGTAGCGGCCACTGATTT
	Probe	6FAM-CCCACACCGGTTTTTCTGTTTTCCAAGTTAA-TAMRA
HERV-W *pol*	Forward	ACMTGGAYKRTYTTRCCCCAA
	Reverse	GTAAATCATCCACMTAYYGAAGGAYMA
	Probe	6FAM-TYAGGGATAGCCCYCATCTRTTTGGYCAGGCA-TAMRA
HERV-H *pol*	Forward	TGGACTGTGCTGCCGCAA
	Reverse	GAAGSTCATCAATATATTGAATAAGGTGAGA
	Probe	6FAM- TTCAGGGACAGCCCTCGTTACTTCAGCCAAGCTC-TAMRA
Syncytin 1 *env*	Forward	ACTTTGTCTCTTCCAGAATCG
	Reverse	GCGGTAGATCTTAGTCTTGG
	Probe	6FAM-TGCATCTTGGGCTCCAT-TAMRA
Syncytin 2 *env*	Forward	GCCTGCAAATAGTCTTCTTT
	Reverse	ATAGGGGCTATTCCCATTAG
	Probe	6FAM- TGATATCCGCCAGAAACCTCCC-TAMRA
TRIM28	Forward	GCCTCTGTGTGAGACCTGTGTAGA
	Reverse	CCAGTAGAGCGCACAGTATGGT
	Probe	6FAM-CGCACCAGCGGGTGAAGTACACC-TAMRA
SETDB1	Forward	GCCGTGACTTCATAGAGGAGTATGT
	Reverse	GCTGGCCACTCTTGAGCAGTA
	Probe	6FAM-TGCCTACCCCAACCGCCCCAT-TAMRA

**Table 2:** Demographics and clinical characteristics of children with acute RSV bronchiolitis. *n*: number; %: percentage; IQR: interquartile range, expressed as 25 and 75 quartile values; yrs: years; SD: standard deviation. Values upper * or below ** normal limit according to age-related cutoffs.

	Patients (n = 37)
Median age (IQR)	0.2 yrs (0.1–0.3)
Males (%)	15 (40.5%)
Comorbidities, n (%)	10 (27%)
Mean interval (+ SD) from symptom onset and sampling	7.0 days (5.5)
Increased inflammatory markers n (%)	10 (27%)
Leukocytosis, n (%) *	3 (13%)
Lymphopenia, n (%) **	8 (21.6%)
Steroid treatment, n (%)	12 (32.4%)
Oxygen treatment (%)	31 (83.8%)

**Table 3:** Characteristics of subgroups of RSV+ patients and healthy children. IQR: interquartile range, expressed as 25 and 75 quartile values.

RSV+ Patients		Healthy Children			
Group A1	Group A2	Group B1	Group B2	Group B3	Group B4
On steroid treatment	No steroid treatment	Tested for interferon signatures	Tested for pol genes of HERV-H, -K, and –W	Tested for env genes of syncytin 1 and syncytin 2	Tested for TRIM28 and SETDB1
n. 12	n. 25	n. 46	n. 29	n. 30	n. 40
7 males, median age 0.29, IQR 0.17–2.25 years	16 males, median age 0.17, IQR 0.11–3.5 years	27 males, median age 0.7, IQR 0.2–2.1 years	17 males, median age 3.3, IQR 2.3–4.1 years	17 males, median age 1.9, IQR 1.1–3.8 years	23 males, median age 3.3, IQR 2.0–3.8 years

## Data Availability

No additional data were created.

## Supplementary Materials

The following supporting information can be downloaded at https://www.mdpi.com/article/10.3390/cimb45020079/s1: Supporting information on the differences between males and females in the transcription levels of all variables studied can be downloaded in the Supplementary Figures S1–S8 at https://www.mdpi.com/. Figure S1. Expression of type I interferon stimulated genes (ISGs) in whole blood from 25 healthy children: 16 males and 9 females. Figure S2. Expression of type I interferon stimulated genes (ISGs) in whole blood from 37 children with acute RSV bronchiolitis: 15 males and 22 females. Figure S3. Expression of type III interferons in whole blood from 25 healthy children: 16 males and 9 females. Figure S4. Expression of type III interferons in whole blood from 37 children with acute RSV bronchiolitis: 15 males and 22 females. Figure S5. Transcription levels of pol genes of HERV-H, HERV-K, and HERV-W in whole blood from 29 healthy children (HC), 17 males and 12 females, and of env genes of syncytin (SYN)1 and syncytin (SYN)2 from 30 HC, 17 males and 13 females. Figure S6. Transcription levels of pol genes of HERV-H, HERV-K, and HERV-W, and of env genes of syncytin (SYN)1 and syncytin (SYN)2 in whole blood from 37 children with acute RSV bronchiolitis: 15 males and 22 females. Figure S7. Expression of TRIM28 and SETDB1 in whole blood from 40 healthy children (HC): 23 males and 17 females. Figure S8. Expression of TRIM28 and SETDB1 in whole blood from 37 children with acute RSV bronchiolitis (RSV+): 15 males and 22 females.

## Abbreviations

HERVs	human endogenous retroviruses
IFN	interferon
ISGs	interferon-stimulated genes
KRAB-ZFPs	Kruppel-associated box domain zinc finger proteins
NS1	RSV-nonstructural protein 1
NS2	RSV-nonstructural protein 2
PICU	pediatric intensive care unit
PBMCs	peripheral blood mononuclear cells
PRR	pattern recognition receptor
RSV	respiratory syncytial virus
SETDB1	domain bifurcated histone lysine methyltrasferase 1
SOCS	suppressor of cytokine signaling
SYN1	syncytin 1
SYN2	syncytin 2
TRIM28	tripartite motif containing 28
TLR	Toll-like receptor
