# Supplementary material for: Expressions of Type I and III Interferons, Endogenous Retroviruses, TRIM28, and SETDB1 in Children with Respiratory Syncytial Virus Bronchiolitis
Source: Curr Issues Mol Biol. 2023 Feb 2;45(2):1197–217. doi: 10.3390/cimb45020079 (PMC9954910; doi:10.3390/cimb45020079)
Supplement: Supplementary file 1 [file cimb-45-00079-s001.zip › cimb-2113757-supplementary.pdf]

Figure 1S. Expression of type I interferon stimulated genes (ISGs) in whole blood from 25 healthy children: 16 males and 9 females.

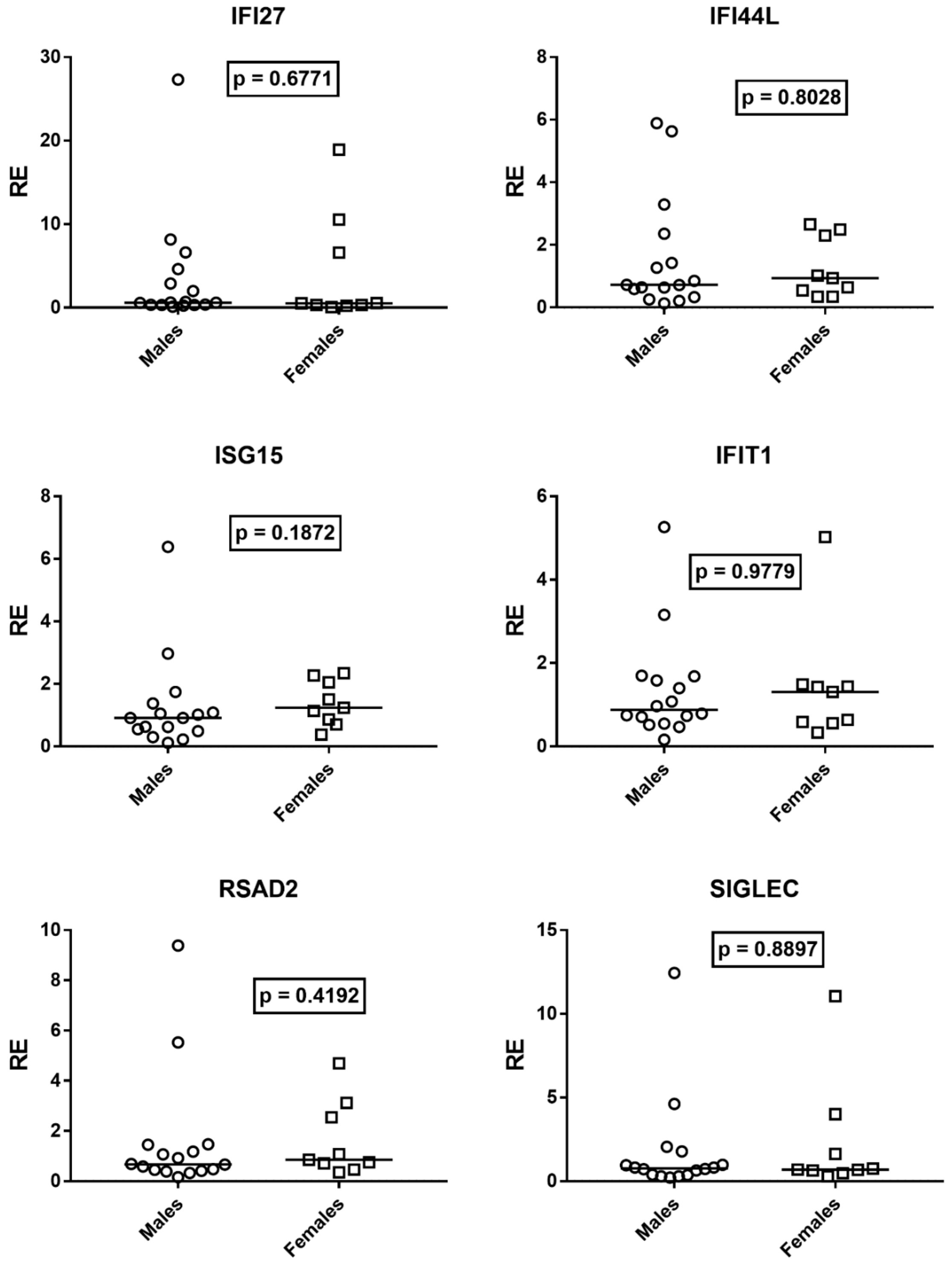

RE: Relative Expression. Circles and squares show the mean of three individual measurements; horizontal lines the median values. Statistical analysis: Mann-Whitney test was used to compare values of males with females. Median values and interquartile range 25%-75% of ISGs: IFI27: Males 0.59, 0.33-3.32; Females 0.51, 0.31-6.59; IFI44L: Males

0.72, 0.53-1.66, Females 0.94, 0.54-2.49; ISG15: Males 0.91, 0.53-1.16; Females 1.23, 0.86-2.05; IFIT1: Males 0.88, 0.67-1.61; Females 1.31, 0.59-1.44; RSAD2: Males 0.67, 0.45-1.25; Females 0.85,0.72-2.54; SIGLEC: Males 0.77, 0.38-1.18; Females 0.70, 0.65-1.63.

Figure 2S. Expression of type I interferon stimulated genes (ISGs) in whole blood from 37 children with acute RSV bronchiolitis: 15 males and 22 females.

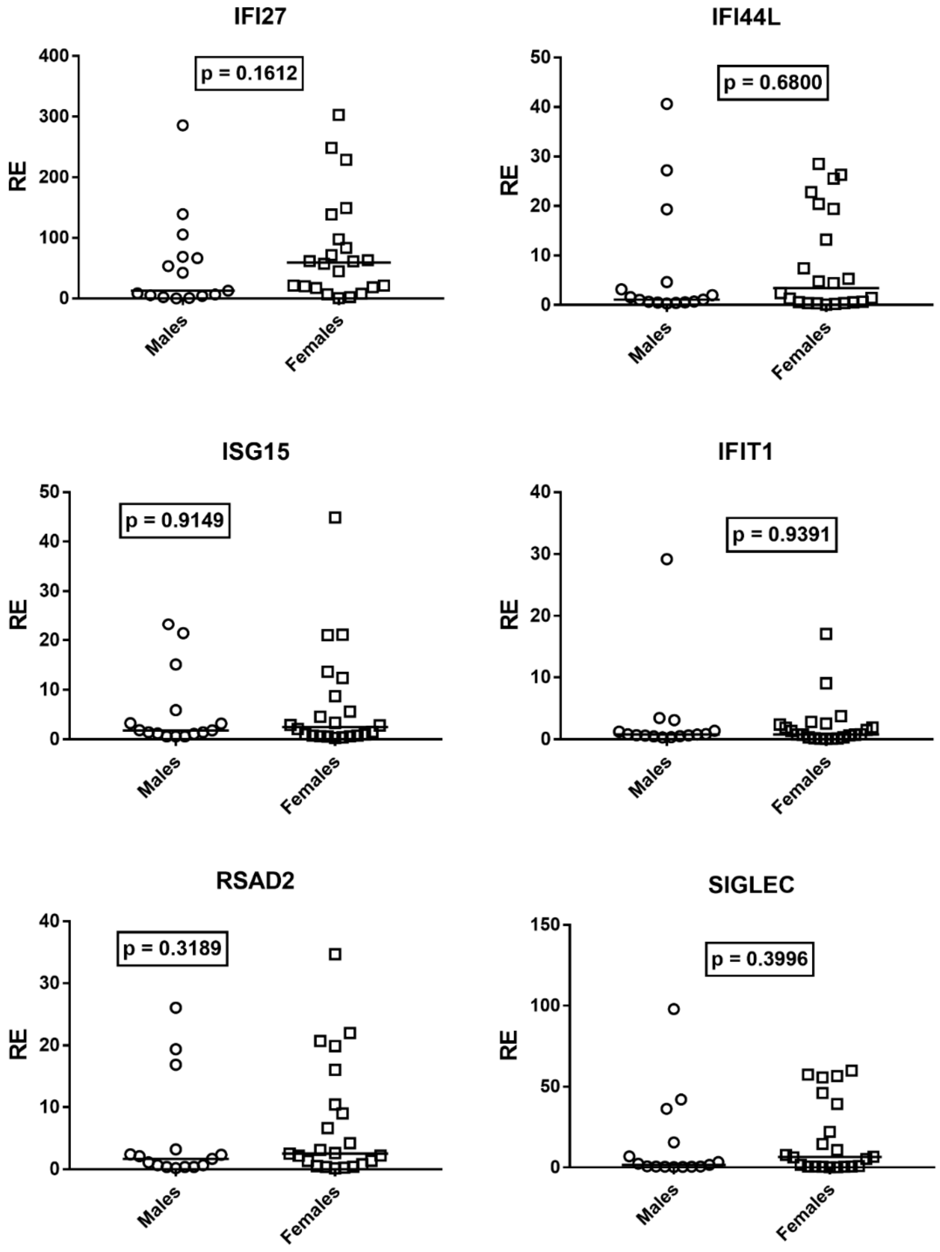

RE: Relative Expression. Circles and squares show the mean of three individual measurements; horizontal lines the median values. Statistical analysis: Mann-Whitney test was used to compare values of males with females. Median values and interquartile range

25%-75% of ISGs: IFI27: Males 12.68, 4.84-67.72; Females 59.14, 18.94-94.19; IFI44L: Male s1.12, 0.62-3.93, Females 3.42, 0.57-17.89; ISG15: Males 1.81, 1.16-4.58; Females 2.47, 0.76-7.94; IFIT1: Males 0.78, 0.56-1.32; Females 0.80, 0.32-2.27; RSAD2: Males 1.69, 0.53-2.82; Females 2.56,0.95-10.09; SIGLEC: Males 1.67, 0.63-11.27; Females 6.50, 0.72-35.04.

Figure 3S. Expression of type III interferons in whole blood from 25 healthy children: 16 males and 9 females.

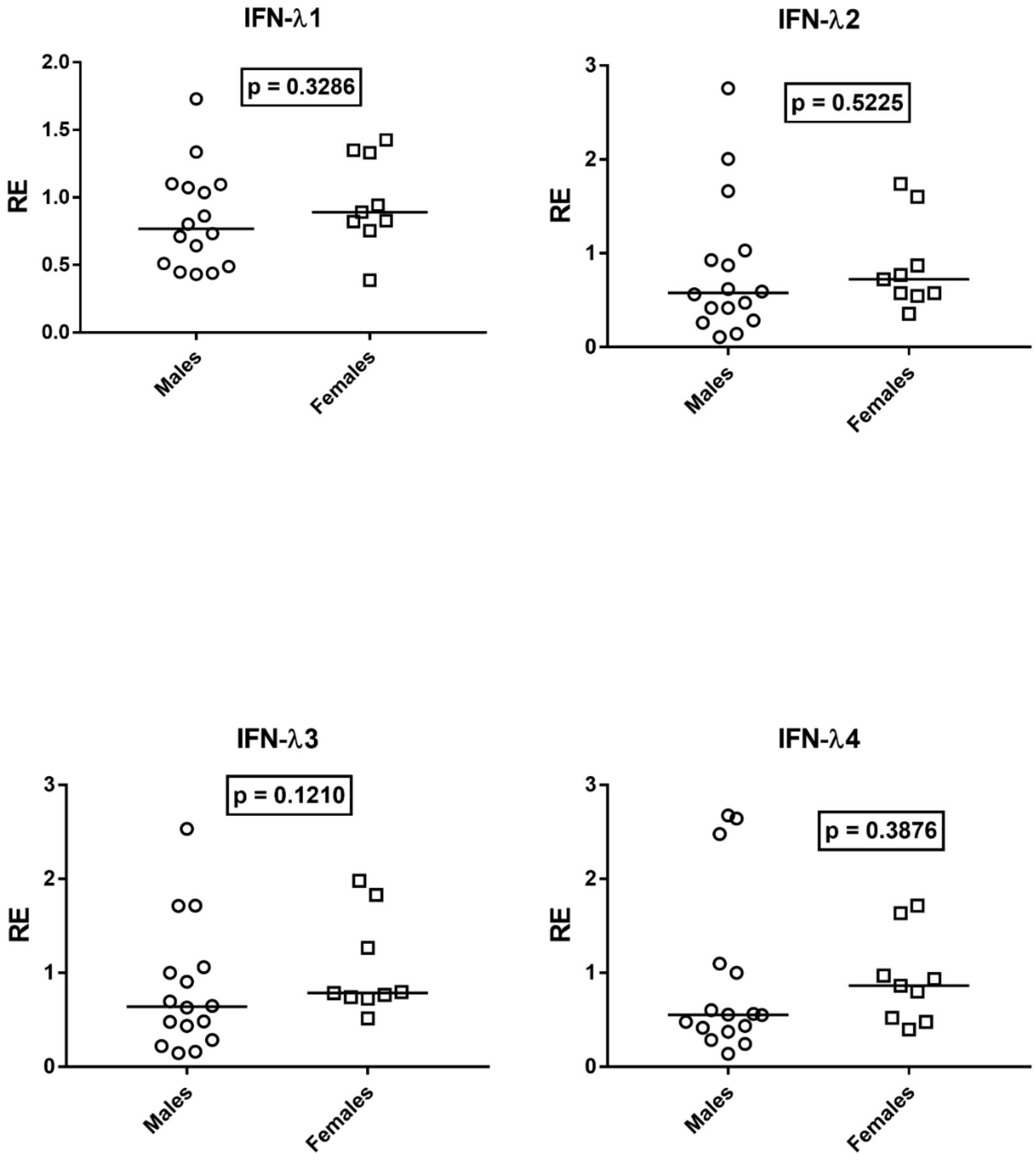

RE: Relative Expression. Circles and squares show the mean of three individual measurements; horizontal lines the median values. Statistical analysis: Mann-Whitney test

was used to compare values of males and females. Median values and interquartile range 25%-75% of IFN-λ1: Male*s* 0.77, 0.51-1.08; Female*s* 0.89, 0.82-1.33; IFN-λ2: Male*s* 0.58, 0.39-0.95; Female*s* 0.72, 0.58- 0.87; IFNλ3: Male*s* 0.64, 0.40-1.02; Female*s* 0.79, 0.74-1.27; IFNλ4: Male*s* 0.55, 0.41-1.03; Female*s* 0.86, 0.52-0.97.

Figure 4S. Expression of type III interferons in whole blood from 37 children with acute RSV bronchiolitis: 15 males and 22 females.

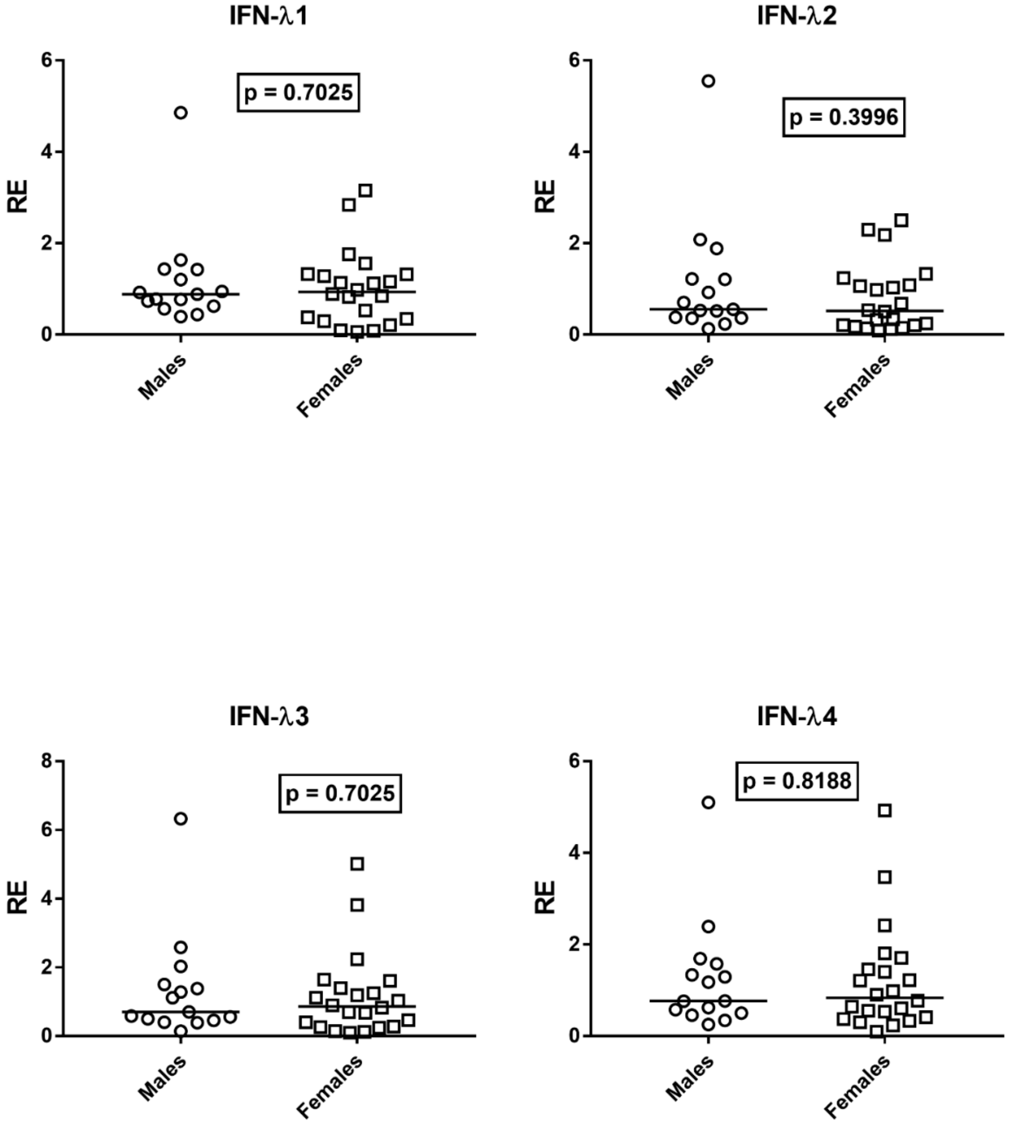

RE: Relative Expression. Circles and squares show the mean of three individual measurements; horizontal lines the median values. Statistical analysis: Mann-Whitney test was used to compare values of males and females. Median values and interquartile range 25%-75%: IFN-λ1: Males 0.89, 0.68-1.32; Females 0.94, 0.36-1.31; IFN-λ2: Males 0.56,

0.38-1.22; Females 0.52, 0.21- 1.08; IFNλ3: Males 0.70, 0.48-1.44; Females 0.87, 0.31-1.36; IFNλ4: Males 0.77, 0.54-1.46; Females 0.84, 0.44-1-45.

Figure 5S. Transcription levels of pol genes of HERV-H, HERV-K, and HERV-W in whole blood from 29 healthy children (HC), 17 males and 12 females, and of env genes of syncytin (SYN)1 and syncytin (SYN)2 from 30 HC, 17 males and 13 females.

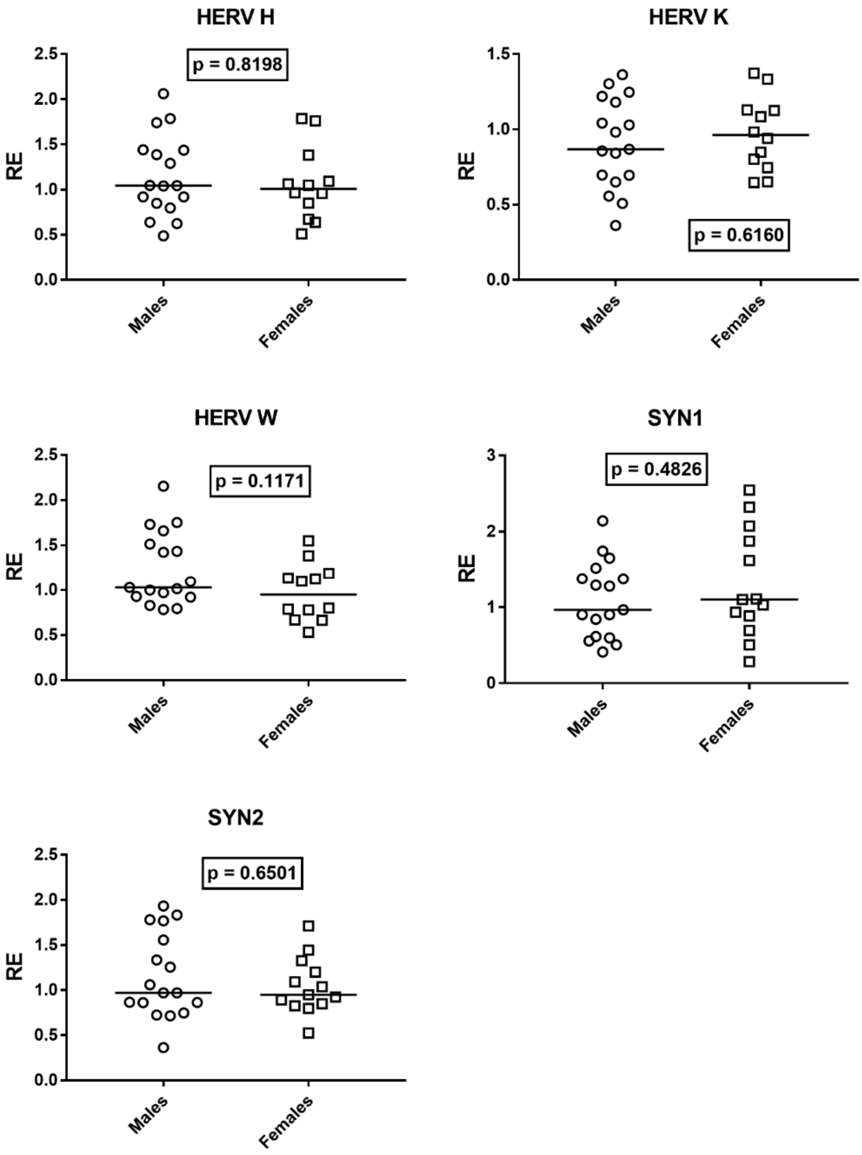

RE: Relative Expression. Circles and squares show the mean of three individual measurements; horizontal lines the median values. Statistical analysis: Mann-Whitney test was used to compare values of males and females. Median values and interquartile range 25%-75%: HERV-H-pol: Male*s* 1.05, 0.85-1.44; Female*s* 1.00, 0.81-1.17; HERV-K-pol:

Male*s* 0.87, 0.70-1.18; Female*s* 0.96, 0.79-1.13; HER-W-pol: Male*s* 1.03, 0.93-1.51; Female*s* 0.95, 0.75-1.15; SYN 1-env: Male*s* 0.97, 0.61-1.38; Female*s* 1.10, 0.89-1.89; SYN2-env: Male*s* 0.97, 0.86-1.56; Female*s* 0.95, 0.85-1.20.

Figure 6S. Transcription levels of pol genes of HERV-H, HERV-K, and HERV-W, and of env genes of syncytin (SYN)1 and syncytin (SYN)2 in whole blood from 37 children with acute RSV bronchiolitis: 15 males and 22 females.

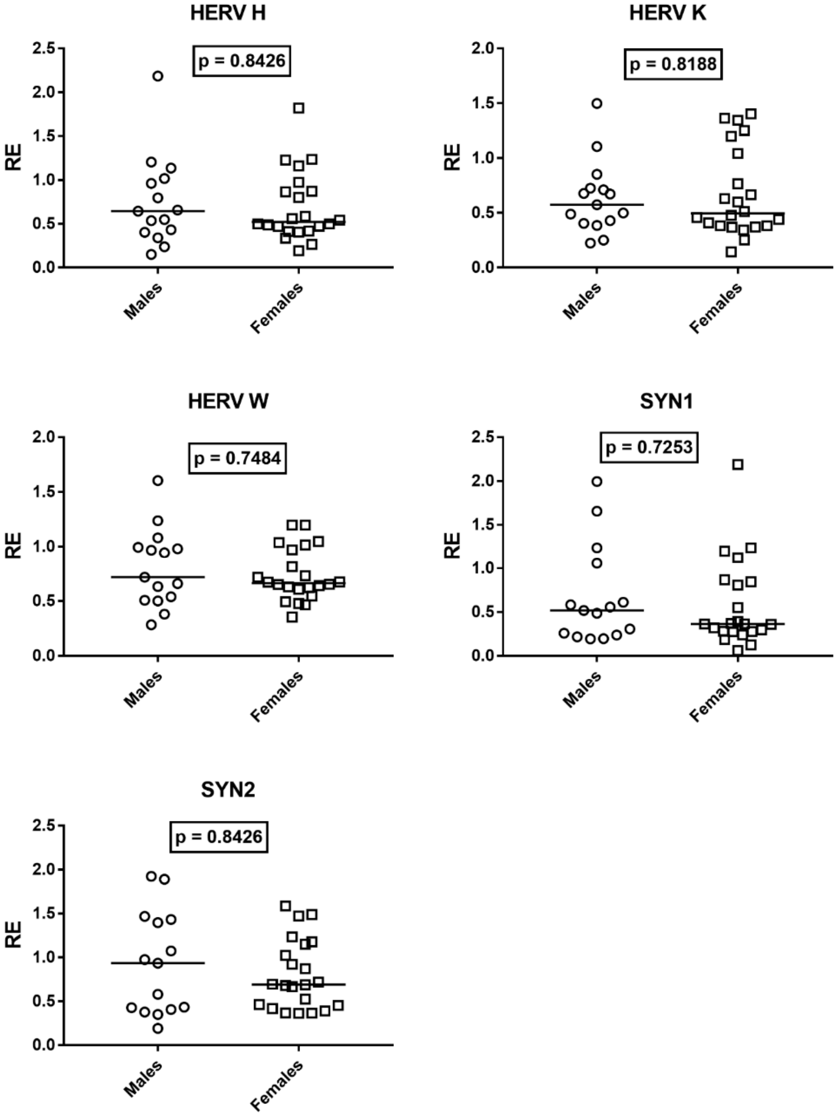

RE: Relative Expression. Circles and squares show the mean of three individual measurements; horizontal lines the median values. Statistical analysis: Mann-Whitney test

was used to compare values of males with females. Median values and interquartile range 25%-75%: HERV-H-pol: Males 0.65, 0.42-1.00; Females 0.52, 0.43-0.87; HERV-K-pol: Males 0.57, 0.42-0.72; Females 0.50, 0.38-0.97; HER-W-pol: Males 0.72, 0.52-0.99; Females 0.66, 0.61-0.93; SYN 1-env: Males 0.52, 0.25-0.83; Females 0.36,0.28-0.84; SYN2-env: Males 0.93, 0.42-1.41; Females 0.69, 0.46-1.12.

Figure 7S. Expression of TRIM28 and SETDB1 in whole blood from 40 healthy children (HC): 23 males and 17 females.

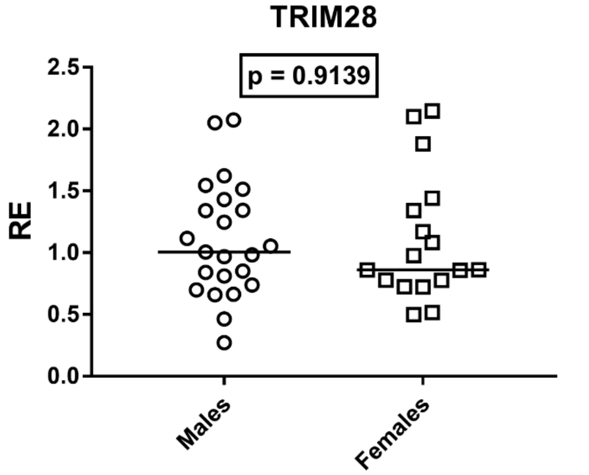

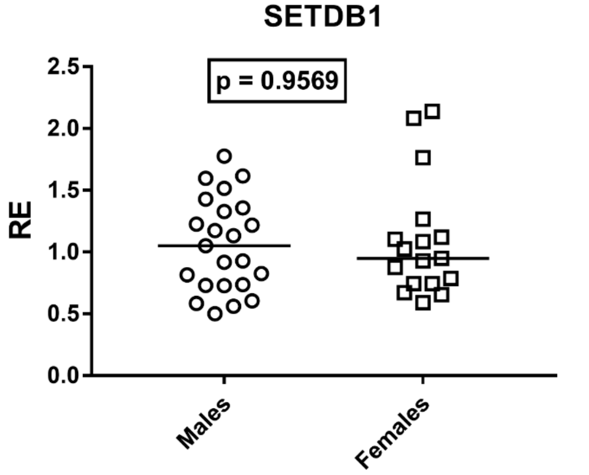

RE: Relative Expression. Circles and squares show the median of three individual measurements, horizontal lines the median values. Statistical analysis: Mann-Whitney test was used to compare values of males with sopra dovrebbero già essere fuori appoggiate sopra Lounge dove c'è la vediamo una libreria in basso sopra la valigia dovrebbe esserci

una scatola E la cosa di quella verde females. Median values and interquartile range 25%-75%: TRIM28: Males 1.00, 0.73-1.34; Females 0.86, 0.78-1.34; SETDB1: Males 1.05, 0.73-1.34; Females 0.95, 0.75-1.12.

Figure 8S. Expression of TRIM28 and SETDB1 in whole blood from 37 children with acute RSV bronchiolitis (RSV+): 15 males and 22 females.

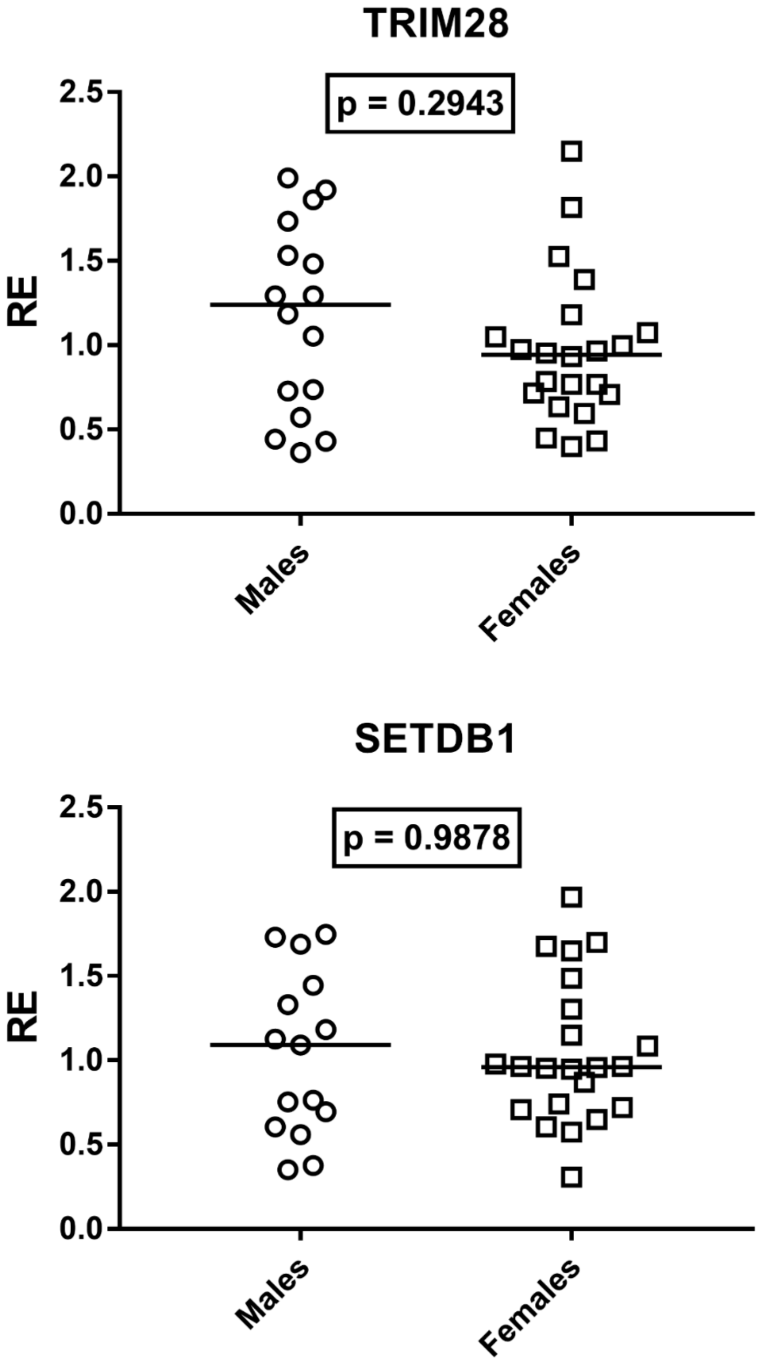

RE: Relative Expression. Circles and squares show the median of three individual measurements, horizontal lines the median values. Statistical analysis: Mann-Whitney test was used to compare values of males with females. Median values and interquartile range 25%-75%: TRIM28: Males 1.18, 0.65-1.63; Females 0.94, 0.71-1.07; SETDB1: Males 1.09, 0.65-1.39; Females 0.96, 0.72-1.27.
